# mRNA-lncRNA Co-Expression Network Analysis Reveals the Role of lncRNAs in Immune Dysfunction during Severe SARS-CoV-2 Infection

**DOI:** 10.3390/v13030402

**Published:** 2021-03-03

**Authors:** Sumit Mukherjee, Bodhisattwa Banerjee, David Karasik, Milana Frenkel-Morgenstern

**Affiliations:** 1 Cancer Genomics and BioComputing of Complex Diseases Lab, Azrieli Faculty of Medicine, Bar-Ilan University, Safed 1311502, Israel; sumit.mukherjee@biu.ac.il; 2 Musculoskeletal Genetics Laboratory, Azrieli Faculty of Medicine, Bar-Ilan University, Safed 1311502, Israel; bodhi.apps@gmail.com (B.B.); david.karasik@biu.ac.il (D.K.)

**Keywords:** lncRNA, co-expression network, COVID-19, cytokine storm

## Abstract

The recently emerged SARS-CoV-2 virus is responsible for the ongoing COVID-19 pandemic that has rapidly developed into a global public health threat. Patients severely affected with COVID-19 present distinct clinical features, including acute respiratory disorder, neutrophilia, cytokine storm, and sepsis. In addition, multiple pro-inflammatory cytokines are found in the plasma of such patients. Transcriptome sequencing of different specimens obtained from patients suffering from severe episodes of COVID-19 shows dynamics in terms of their immune responses. However, those host factors required for SARS-CoV-2 propagation and the underlying molecular mechanisms responsible for dysfunctional immune responses during COVID-19 infection remain elusive. In the present study, we analyzed the mRNA-long non-coding RNA (lncRNA) co-expression network derived from publicly available SARS-CoV-2-infected transcriptome data of human lung epithelial cell lines and bronchoalveolar lavage fluid (BALF) from COVID-19 patients. Through co-expression network analysis, we identified four differentially expressed lncRNAs strongly correlated with genes involved in various immune-related pathways crucial for cytokine signaling. Our findings suggest that the aberrant expression of these four lncRNAs can be associated with cytokine storms and anti-viral responses during severe SARS-CoV-2 infection of the lungs. Thus, the present study uncovers molecular interactions behind the cytokine storm activation potentially responsible for hyper-inflammatory responses in critical COVID-19 patients.

## 1. Introduction

COVID-19 is a severe acute respiratory disease caused by SARS-CoV-2, a recently identified member of the Coronaviridae family [1,2]. The number of cases and deaths due to COVID-19 is increasing daily and threatens global health. While several drugs and vaccines are under clinical trial, to date, there is no effective strategy to control the COVID-19 disease [3,4,5]. The clinical spectrum of COVID-19 is variable and can lead to immune dysfunction, acute respiratory distress syndrome (ARDS), and multi-organ failure [6,7,8]. Human coronaviruses, including SARS-CoV, MERS-CoV, and SARS-CoV-2, have evolved strategies to delay or dampen interferon production, which sometimes produces host inflammatory responses leading to ARDS [9,10]. Dysregulated host immune responses and increased circulatory levels of pro-inflammatory cytokines, a phenomenon known as a “cytokine storm,” is strongly associated with lung injury, multi-organ failure, and poor prognosis of severe COVID-19 cases [11,12,13]. Recent studies demonstrated that patients afflicted with severe SARS-CoV-2 infections present increased levels of pro-inflammatory plasma cytokines, as opposed to mild cases, highlighting that the release of inflammatory cytokines is central to COVID-19 severity [14,15,16]. The generation of pro-inflammatory cytokines in severe COVID-19 infection is characterized by high expression of IL-6 and TNF-α [17]. When SARS-CoV-2 enters respiratory epithelial cells, the host immune response is activated by TNF-α, which mediates cytokine and chemokine production, leading to systemic inflammatory responses [18]. Similarly, SARS-CoV-2 infection induces higher expression of IL-6, which inhibits natural killer (NK) cell-mediated cytotoxicity and down-regulates perforin and granzyme B expression, leading to the failure of NK cells to destroy target cells [17,19]. Therefore, the survival of target cells facilitates enhancing antigen stimulation and promotes the overproduction of pro-inflammatory cytokines. Dysregulation of cytokine production elicits the delivery of various immune cells, such as macrophages, neutrophils, and T cells, to the infection site to generate an inflammatory response. This has a destructive effect on lung tissues, leading to ARDS [20]. However, the underlying molecular mechanisms responsible for the aberrant inflammatory responses and immune dysfunction seen during severe cases of COVID-19 are still unknown.

Immune cell development and activation depend on gene expression’s dynamic regulation through complex transcriptional and post-transcriptional mechanisms [21]. Long non-coding RNAs (lncRNAs) are transcripts 200 nucleotides or longer in length, which play pivotal roles in regulating diverse biological processes [22]. Multiple studies based on genome-wide association studies (GWAS) have identified numerous single nucleotide polymorphisms (SNPs) within various dysregulated lncRNAs associated with different human diseases [23]. Indeed, lncRNAs have emerged as potential key regulators of inflammatory genes and serve vital roles in regulating inflammatory responses [24,25,26]. For instance, recent studies demonstrated that lncRNAs are involved in regulating gene expression in immune cells [27]. However, those mechanisms employed by lncRNAs to regulate immune function remain largely unknown.

Given the role(s) of lncRNAs in host cell anti-viral inflammatory response regulation, we sought to identify lncRNAs that are co-expressed with human genes involved in immune-related processes during SARS-CoV-2 infection in the lungs. We thus identified common differentially expressed (DE) mRNAs and lncRNAs from various publicly available SARS-CoV-2 infected lung transcriptome datasets. We subsequently identified a key lncRNA-mRNA module enriched for elements of different immune-related pathways related to cytokine signaling based on weighted gene co-expression analysis (WGCNA). Network analysis also revealed four lncRNAs as potential hubs, thereby pointing to the possible association of these lncRNAs with cytokine signaling during SARS-CoV-2 infection and potential involvement in hyper-inflammatory responses during SARS-CoV-2 infection of the lungs. These findings could thus advance understanding of complex interactions behind the immune dysfunction experienced in severe cases of COVID-19. 

## 2. Materials and Methods

### 2.1. Acquisition of Transcriptome Dataset

Several sets of raw sequence reads were retrieved from Gene Expression Omnibus (GEO) datasets. GEO dataset GSE147507 contains data from cell lines infected with SARS-CoV-2, including Calu-3 adenocarcinoma cells and A549 cells supplemented with a vector expressing ACE2. GEO dataset GSE139516 contains data from the Calu3 cell line infected with Middle East respiratory syndrome coronavirus (MERS-CoV), while GEO dataset GSE148729 contains data from the Calu3 cell line infected with SARS-CoV-1 and SARS-CoV-2. Raw sequence data from BALF of COVID-19 patients were retrieved from the Genome Sequence Archive (https://bigd.big.ac.cn/, accessed on 2 February 2021), Beijing Institute of Genomics (accession number CRA002390) [28]. RNA-seq data for BALF from healthy control samples were downloaded from the NCBI SRA database (accession numbers SRR10571724, SRR10571730, and SRR10571732). Data from mock-treated cells were provided for each in vitro group (*N* = 3 per group). The raw reads were retrieved and converted to FASTQ using the SRA toolkit, version 2.10.7. 

### 2.2. Read Mapping and Differential Expression Analysis

Raw reads were aligned to the latest human genome build (GRCh38.p13), using reference annotations derived from GENCODE release 35 by STAR, v2.7.5 [29]. To increase alignment specificity, STAR was run in the 2-pass mode using the “—twopassMode” basic command. The transcripts were assembled with StringTie, v2.1.2 [30] using the reference annotation. Raw read counts of the mRNAs and annotated lncRNAs were determined by featureCounts, while differential expression analysis was performed using edgeR [31]. The Benjamini–Hochberg method for controlling the false discovery rate (FDR) was used for adjusting *p*-values. Genes with FDR < 0.05 and fold-change ≥ 2 were considered as being differentially expressed.

### 2.3. Co-Expression Network Analysis

WGCNA approaches [32] were used to determine the correlation between DE lncRNAs and mRNAs. The WGCNA method identifies network modules consisting of highly correlated genes in terms of expression that are likely to be involved in the same biological process(es). Co-expression analysis was performed as follows. Initially, a co-expression network was constructed by evaluating the connection strength between mRNAs and lncRNAs. Next, co-expression modules were identified based on hierarchical clustering and dynamic tree cut. Finally, hub nodes were identified in the co-expression module. Cytoscape 3.8.0 (http://www.cytoscape.org/, accessed on 2 February 2021) [33] was used to visualize mRNA and lncRNA co-expression networks in WGCNA modules. The cytoHubba [34] plug-in of Cytoscape was used to identify hub nodes from the co-expression modules. The top ten hub nodes were ranked according to the method of maximal clique centrality (MCC) [35]. 

### 2.4. Gene Ontology and Pathway Enrichment Analysis

After identifying the different modules in the co-expression network, gene set enrichment and pathway enrichment analysis were performed to explore functional categories associated with modules. Gene ontology (GO) and pathway enrichment analyses were performed using ShinyGO [36], Metascape [37], and Parametric Gene Set Enrichment Analysis (PGSEA) [38] with a *p*-value cutoff <0.05. KEGG and REACTOME pathway databases were consulted during pathway enrichment analysis. Fisher’s exact test followed by Benjamini–Hochberg multiple testing correction was employed for selecting the GO category and the relevant pathway. 

## 3. Results

### 3.1. Identification of Common DE mRNAs and lncRNAs in Response to SARS-CoV-2 Infection of the Lungs

**Figure 1:**
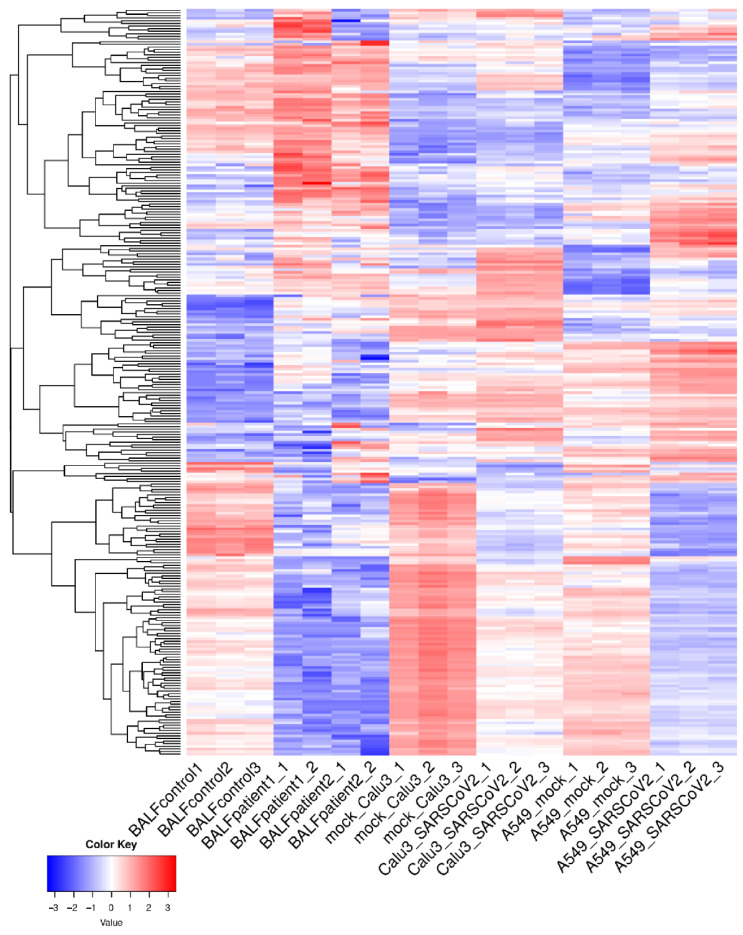
Hierarchical clustering with Euclidean distance of common differentially expressed genes and lncRNAs across all samples.

To understand host transcriptional dynamics in response to SARS-CoV-2 infection of the lungs, we employed the publicly available RNA-seq datasets from BALF samples from COVID-19 patients [28] and SARS-CoV-2-infected lung epithelium cell lines (Calu3 and A549-ACE2) [39]. DE genes from each individual datasets (FDR < 0.05; fold-change ≥ 2) were identified. Protein-coding genes and lncRNAs found to be consistently up-/down-regulated in all individual datasets were selected for further analysis. This revealed 285 common DE genes that included 12 lncRNAs which were consistently up-/down-regulated in BALF samples from COVID-19 patients and in the SARS-CoV-2-infected lung epithelium cell lines (Table S1). Average-linkage hierarchical clustering was performed, applying Euclidian distances for the 285 genes of interest. A detailed representation of the sample cluster dendrogram is presented in Figure 1. 

**Figure 2:**
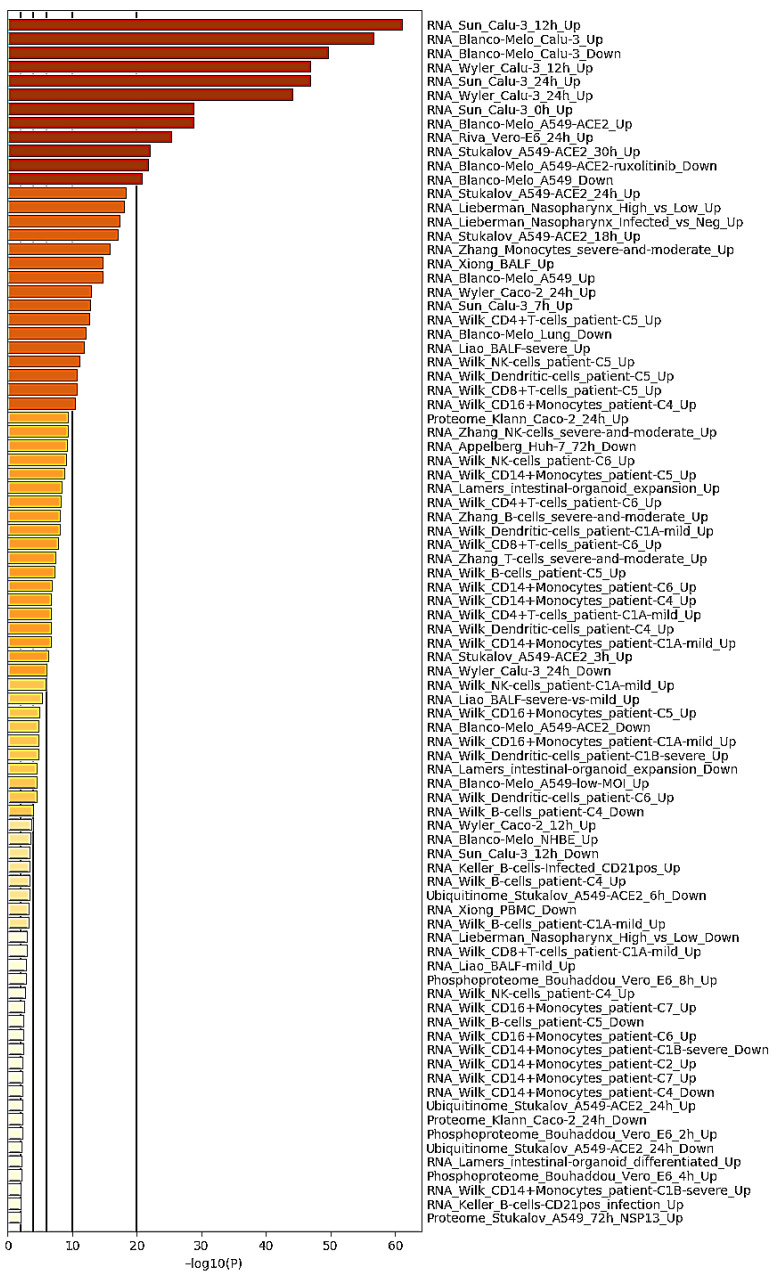
Summary of gene enrichment in COVID-19 analysis. Terms with a *p*-value < 0.01, a minimum count of 3, and an enrichment factor >1.5 (the enrichment factor is the ratio between the observed counts and counts expected by chance) were collected and grouped into clusters based on membership similarities.

Gene set enrichment analysis was next conducted using Coronascape (a specialized version of the Metascape platform (http://coronascape.org, accessed on 2 February 2021)) [37] to reveal expression patterns of the common DE genes identified in transcriptomic data of other available SARS-CoV-2-infected samples. Such analysis showed that a significant proportion of genes and lncRNAs were consistently differentially expressed in COVID-19 patient-derived samples and various SARS-CoV-2-infected cell lines (Figure 2).

### 3.2. Pathway Enrichment Analysis of Common DE Genes Highlight Potential Roles in Cytokine Signaling

**Figure 3:**
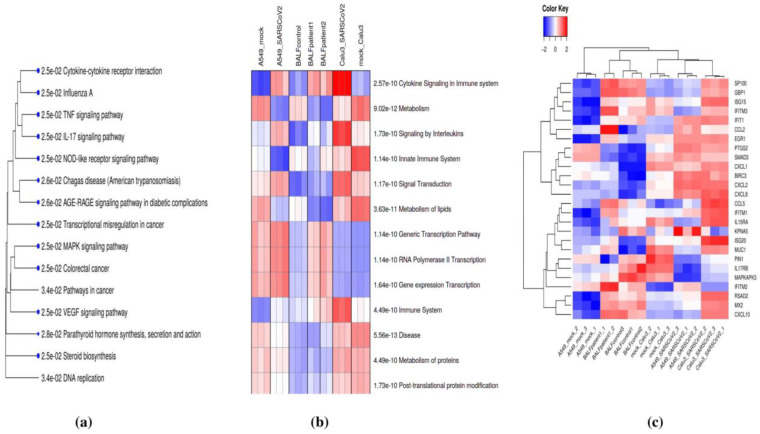
(**a**) Pathway enrichment analysis of common differentially expressed (DE) genes using the KEGG database. (**b**) Parametric Gene Set Enrichment Analysis (PGSEA) analysis of common DE genes using the Reactome database. Red and blue indicate activated and suppressed pathways, respectively. (**c**) Patterns of expression of genes involved in cytokine signaling in the immune system in SARS-CoV-2-infected and control datasets.

Genes involved in the same pathway or that participate in similar biological processes often present correlated gene expression patterns [40]. To identify those pathways most altered upon SARS-CoV-2 infection of the lungs, pathway enrichment analysis was performed based on ShinyGO [36] and PGSEA [38] using the Reactome and KEGG databases with an FDR cutoff of 0.05. Pathway enrichment analysis using the KEGG database highlighted the set of DE genes enriched for several immune-related pathways, including cytokine–cytokine receptor interactions, TNF signaling, and the IL-17 pathway, all of which are crucial for the production of various inflammatory cytokines (Figure 3a). Pathway analysis using the Reactome database revealed that cytokine signaling in immune systems (R-HSA-1280215) was the most enriched pathway that was consistently up-regulated in all SARS-CoV-2-infected cell samples and COVID-19 patient samples (Figure 3b. Further analysis of the expression patterns of DE genes associated with cytokine signaling processes across all samples revealed most of these genes to be up-regulated in SARS-CoV-2-infected samples compared to control samples (Figure 3c). These findings thus elucidate gene expression patterns involved in activating cytokine signaling during SARS-CoV-2 infection of the lungs.

### 3.3. Co-Expression Analysis and Identification of a Key Module Associated with Cytokine Signaling 

**Figure 4:**
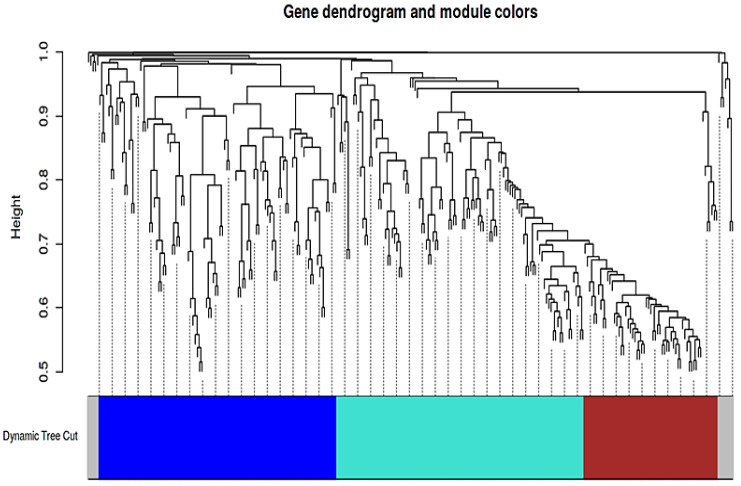
Cluster dendrogram and module assignment for modules from WGCNA. Branches of the dendrogram group contain densely interconnected, highly co-expressed genes and lncRNAs.

To identify potential lncRNA-mRNA interaction networks among DE genes crucial for cytokine signaling during SARS-CoV-2 infection, co-expression analysis based on WGCNA was performed [32]. Module detection analysis of the common DE datasets consisting of mRNA and lncRNAs was initially conducted. Using WGCNA [32], the soft threshold power of β was 12 when the scale-free topology model fit R^2^ was maximized. Co-expression modules were then determined by the dynamic tree cut procedure using the dynamic branch-cutting algorithm with a robust measure of interconnectedness, using DynamicTreeCut and the WGCNA R library. A total of three modules were identified in the network, with each module being assigned a unique color label (blue, brown, or turquoise), as represented in Figure 4. In the blue module, four lncRNAs that produce a co-expression network of 105 protein-coding genes were found. In the turquoise module, eight lncRNAs that co-expressed with 104 protein-coding genes were detected. Sixty-three protein-coding genes were found to be co-expressed in the brown module, which did not contain lncRNAs.

**Figure 5:**
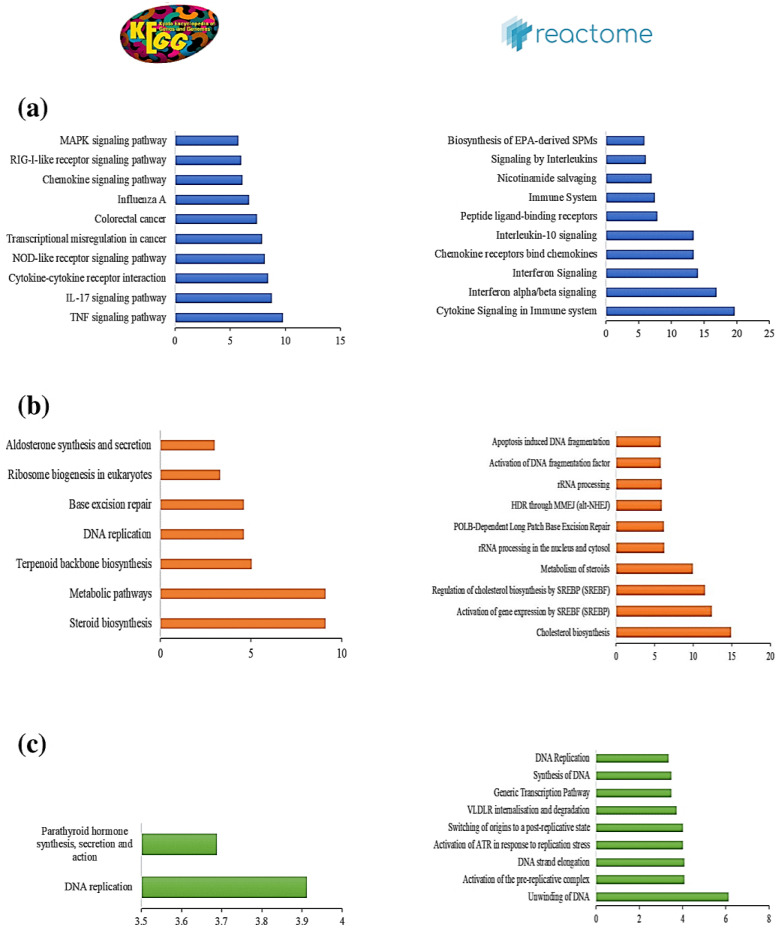
The ten most-highly enriched KEGG and Reactome pathways in the (**a**) blue, (**b**) brown, and (**c**) turquoise modules.

To explore the biological functions of the three modules, functional enrichment analysis based on GO biological processes and the KEGG and Reactome pathways was performed using ShinyGO [36] (with an FDR cutoff of 0.05) (Table S2). The results of the topmost enriched pathways for each module are depicted in Figure 5. Interestingly, we found only the blue module to be enriched in genes for several immune-related processes and pathways associated with cytokine and interferon signaling and involved in the TNF, IL-17, and NF-kappa B signaling pathways. This indicates that the co-expression network based on this module might be associated with the cytokine storms during severe SARS-CoV-2 infection of the lungs.

### 3.4. Analysis of Hub Nodes from lncRNA-mRNA Co-Expression Networks Reveals the Potential Involvement of lncRNAs in Cytokine Signaling

**Figure 6:**
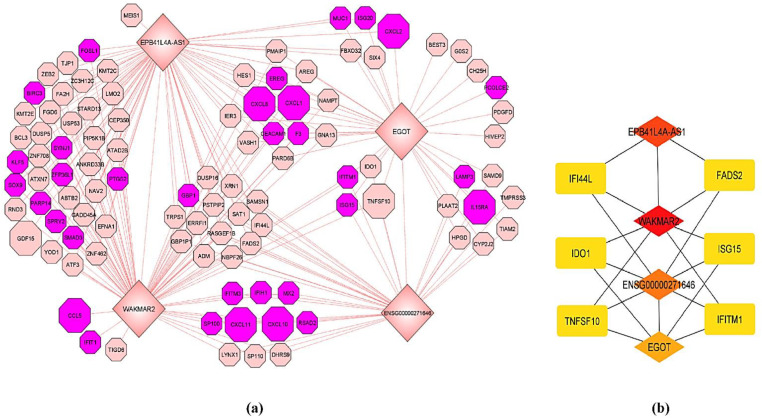
(**a**) mRNA-lncRNA co-expression network of the blue module. lncRNAs are represented by diamond shapes. The genes highlighted in purple belong to the enriched biological process “response to cytokines.” The genes represented by large octagon shapes represent the enriched KEGG pathway “cytokine-cytokine receptor interaction.” (**b**) Sub-network of the top ten hub nodes. Increasingly red color represents higher-scoring nodes.

Based on the above analysis and considering the importance of the blue co-expression module for cytokine signaling, a sub-network based on this co-expression module was constructed. Four lncRNAs (WAKMAR2, EGOT, EPB41L4A-AS1, and ENSG00000271646) in this module were significantly up-regulated in the SARS-CoV-2-infected samples. To understand the lncRNAs expression profiles and assess their relationships with protein-coding genes within this module, the co-expression network was constructed, and hub nodes in this module were analyzed. A sub-network of the four lncRNAs and 105 protein-coding genes which were strongly co-expressed in the blue module, was assembled (Figure 6a). Those nodes which are highly connected and essential in this sub-network were identified using the cytoHubba plug-in [34] of Cytoscape [33]. The top 10 hub nodes were identified based on the MCC algorithm. According to the MCC algorithm ranking, the four lncRNAs were the top hub nodes (Figure 6b). To further verify the MCC algorithm results, hub node analysis was repeated using two other popular topological analysis methods: maximum neighborhood component (MNC) analysis and the density of maximum neighborhood component (DMNC) analysis. As with the MCC algorithm, such efforts also found the four lncRNAs to be the top-ranking hub nodes. As such, it would appear that these four lncRNAs are associated with cytokine signaling during severe SARS-CoV-2 infection of the lungs.

### 3.5. Expression Profiling of lncRNAs and Their Interactors in the SARS and MERS-Infected Calu3 Cell Line

We analyzed the expression patterns of four lncRNAs (WAKMAR2, EGOT, EPB41L4A-AS1, and ENSG00000271646) and their interactors in the SARS and MERS-infected Calu3 cell line to understand the degree of similarity of interactions mediated by these lncRNAs during other human coronavirus infections. We observed that among these four lncRNAs, EGOT was found to be significantly differentially expressed in both SARS (logFC = 3.216, FDR = 3.2 × 10^−30^) and MERS-infected (logFC = 5.547, FDR = 9.6 × 10^−210^) Calu3 cells. WAKMAR2 was found to be differentially expressed only in MERS-infected (logFC = 4.846, FDR = 6.06 × 10^−30^) Calu3 cells, while ENSG00000271646 was found to be differentially expressed only in SARS-infected (logFC = 1.815, FDR = 2.05 × 10^−7^) Calu3 cells. Furthermore, we analyzed how many interactors of these four lncRNAs from the co-expression network derived from the blue module are also differentially expressed in SARS and MERS-infected cells. Among the 105 protein-coding interactors from the blue module, we observed 74 DE protein-coding genes in SARS-CoV-infected cells and 58 DE protein-coding genes in MERS-infected cells (Table S3). This analysis indicates that SARS-CoV-2 evolved a different strategy to induce the host immune response, different from other previous human coronavirus infections.

## 4. Discussion

The dysregulated release of pro-inflammatory cytokines referred to as a “cytokine storm” has been reported as one of the critical factors behind poorer outcomes resulting from COVID-19 [12,16,41,42]. A cytokine storm produces an aberrant inflammatory and immune response in the lungs, resulting in acute respiratory distress, pulmonary edema, and multi-organ failure [12,17]. Yet, the host factors required for the SARS-CoV-2 propagation in the lungs and the underlying molecular mechanisms for cytokine storm generation during severe SARS-CoV-2 infection remain unknown. 

Analysis of co-expression networks provides an essential framework for identifying sets of protein-coding genes and lncRNAs that respond to a pathogenic condition in a coordinated manner, highlighting their potential regulatory relationship. To understand host transcriptional dynamics in dysregulated immune system function in severe SARS-CoV-2-mediated lung infection, co-expression networks involving common differentially expressed mRNAs and lncRNAs from BALF samples of COVID-19 patients [28], and expression data of SARS-CoV-2-infected human lung epithelium cell lines were analyzed [39]. Using the WGCNA approach [32], a key module consisting of four lncRNAs and 105 mRNAs enriched for different immune-related processes related to cytokine and interferon signaling was identified. The top three enriched KEGG pathways associated with this key module were TNF signaling, IL-17 signaling, and cytokine–cytokine receptor interactions. Recent studies demonstrated that severe cases of COVID-19 exhibit increased plasma levels of TNF-α and IL-17 compared to mild cases [43,44]. Both TNF and IL-17 signaling are associated with activation of the nuclear factor κB (NF-κB) pathway, leading to the release of various inflammatory cytokines that generate the cytokine storm seen during severe COVID-19 infection [41,45,46]. Previous studies demonstrated that the SARS-CoV spike protein induces shedding of the extracellular ACE2 receptor domain, resulting in a loss of ACE2 function and production of TNF-α, which stimulates the NF-κB pathway [47]. Other studies of SARS-CoV-2 showed that the combined production of TNF-α and IFN-γ specifically induced inflammatory cell death and associated lung damage [48]. However, the molecular interactions behind the dysregulation of the TNF and IL-17 signaling pathways seen in severe cases of COVID-19 are still unknown. 

Hub node analysis was conducted to identify the top nodes in the co-expression network, which are strongly associated with the aberrant release of pro-inflammatory cytokines and dysregulation of immune systems during SARS-CoV-2 infection in the lungs. Such analysis revealed that all of the four lncRNAs in the WGCNA-identified module (WAKMAR2, EGOT, EPB41L4A-AS1, and ENSG00000271646) correspond to the top nodes in this sub-network. This finding suggests that these four lncRNAs are strong interactors of cytokine-related genes in the network and their potential interactions are crucial for generating dysfunctional immune responses. Recent studies demonstrated that low expression of WAKMAR2 up-regulates NF-κB signaling and enhances inflammatory cytokine production by keratinocytes in chronic wounds [49]. In response to SARS-CoV-2 infection of lung cells, a consistently higher expression of WAKMAR2 was noted, suggesting that aberrant cytokine release could be the major driver of higher WAKMAR2 expression, which could in turn negatively affect the anti-viral response. Similarly, the EGOT lncRNA was also consistently up-regulated in SARS-CoV-2-infected samples. Previous studies showed that EGOT is induced by NF-κB and functions as a negative regulator of the type I interferon response [50]. Therefore, increased expression of WAKMAR2 and EGOT might favor viral replication in SARS-CoV-2-infected cells. However, the roles of the other two lncRNAs (EPB41L4A-AS1 and ENSG00000271646) in anti-viral responses and cytokine signaling are not known. Since lncRNAs are involved in epigenetic and post-transcriptional regulation, we hypothesize that aberrant cytokine release is the driver for up-regulation of these four lncRNAs, which could negatively affect anti-viral responses that might lead to immune dysfunction during severe COVID-19 infection. 

Overall, our results suggest that these four DE lncRNAs are associated with cytokine signaling and hyper-inflammatory responses during severe SARS-CoV-2 infection of the lungs. Furthermore, our study reveals that the molecular mechanisms of dysregulated immune responses due to the SARS-CoV-2 infection are different from SARS and MERS, leading to the severity of the response to SARS-CoV-2. This study reveals potential associations of lncRNAs in cytokine signaling during the response to severe SARS-CoV-2 infection in the lungs, which indicates the translational potential of those lncRNAs. Our analysis provides novel insight in the search for potential mechanisms underlying immune dysfunction in response to cytokine storms during severe SARS-CoV-2 infection and could also shed light on how cytokine inhibitors could be useful for treating acute cases of COVID-19.

## 5. Conclusions

The results reported here provide novel transcriptomic insight into host responses upon severe SARS-CoV-2 infection. Comprehensive lncRNA and mRNA transcriptomes in SARS-CoV-2-infected human lung epithelial cell lines and BALF from COVID-19 patients were profiled. Through co-expression network analysis, four DE lncRNAs were identified as hub nodes that strongly correlated to the protein-coding genes in this network and enriched for different immune-related processes related to cytokine and interferon signaling. These findings reveal the potential role of lncRNAs in regulating anti-viral responses during severe SARS-CoV-2 infection. This study could thus provide valuable transcriptomic insight into pro-inflammatory cytokine production and the hyper-inflammation caused by severe SARS-CoV-2-induced infection of the lungs. Still, further experimental studies are necessary to elucidate the specific functions of these lncRNAs in COVID-19 pathogenesis.

## Data Availability

Not applicable.

## Supplementary Materials

The following are available online at https://www.mdpi.com/1999-4915/13/3/402/s1, Table S1: Common DE genes which are consistently up-/down-regulated in BALF samples from COVID-19 patients and in the SARS-CoV-2-infected lung epithelium cell lines, Table S2: GO biological processes and pathway enrichment analysis for co-expression modules, Table S3: Differentially expressed mRNAs and lncRNAs in SARS and MERS infected Calu3 cell-line.
